# Ein heller Fleck beim Lesen

**DOI:** 10.1007/s00347-023-01850-4

**Published:** 2023-04-18

**Authors:** Louisa Farghaly, Matthias Grassmann

**Affiliations:** Helios Klinikum Krefeld, Augenklinik, Lutherplatz 40, 47805 Krefeld, Deutschland

## Anamnese

Im April 2022 stellte sich erstmals eine 56-jährige Patientin in unserer Klinik vor. Sie beschrieb eine seit 5 bis 6 Wochen persistierende Sehverschlechterung des rechten Auges. Vor allem beim Lesen sei sie durch einen „hellen Fleck“ am rechten Auge gestört, sodass sie die Anfangsbuchstaben der Worte schlecht erkenne. Am linken Auge wurden keine Beschwerden angegeben. Ophthalmologische Vorerkrankungen seien nicht bekannt. Aufgrund eines beidseitigen Mammakarzinoms erhalte die Patientin eine Immuntherapie mit Trastuzumab und Pertuzumab. Ende Februar 2022 erfolgte eine Impfung mit Comirnaty® von BioNTech/Pfizer gegen COVID-19. Eine COVID-19-Infektion sei anamnestisch nicht bekannt.

## Befund

**Figure 1:**
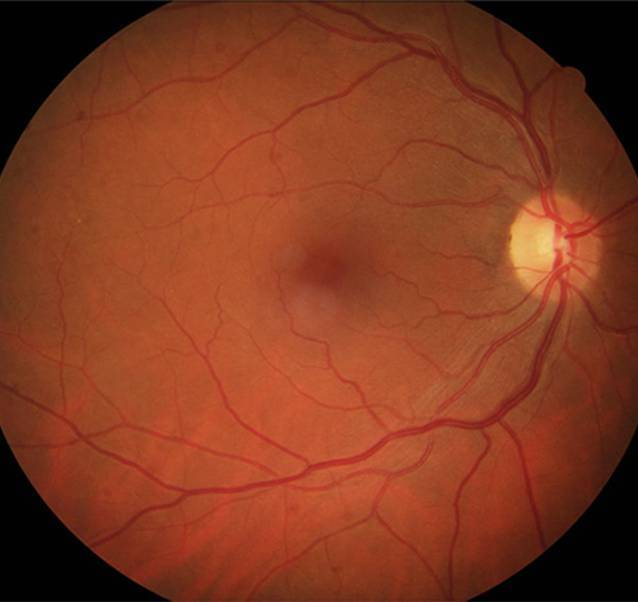

In der klinischen Untersuchung ergab sich ein bestkorrigierter dezimaler Fernvisus von 0,6 am rechten Auge sowie von 0,8 am linken Auge. Im Amsler-Test gab die Patientin nur mit dem rechten Auge Metamorphopsie knapp parazentral an. Der vordere Augenabschnitt war bis auf oberflächige Vaskularisationen der Hornhaut des linken Auges bei Kontaktlinsengebrauch beidseits unauffällig. Fundoskopisch zeigte sich rechts ein stumpf wirkender Reflex der Fovea, sonst beidseits keine Auffälligkeiten (Abb. 1).

**Figure 2:**
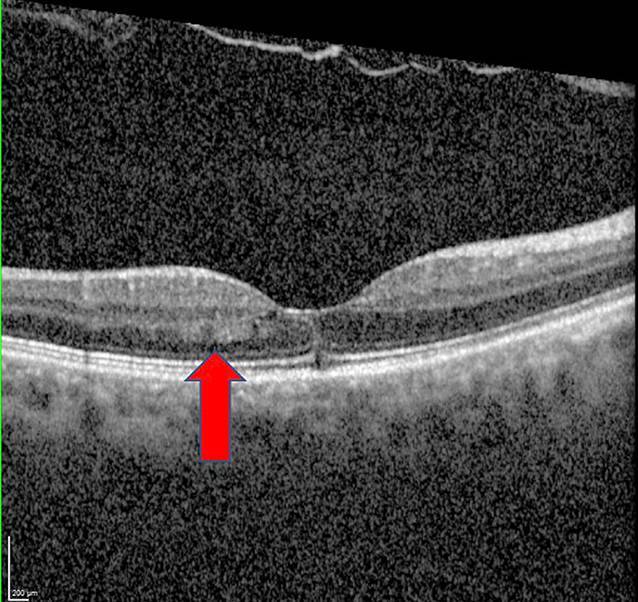

**Figure 3:**
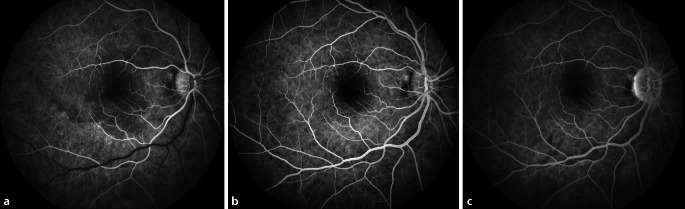

Die optische Kohärenztomographie (OCT) der Makula des linken Auges war unauffällig. Am rechten Auge fiel eine Signalanhebung der äußeren plexiformen und äußeren Körnerschicht temporal der Fovea auf. Die ellipsoide Zone und die IS/OS-Junctions stellten sich regelrecht dar (Abb. 2). Die Fluoreszenzangiographie und die Perimetrie ergaben einen unauffälligen Befund (Abb. 3).

## Diagnose

In Zusammenschau von Symptomatik, Untersuchungsbefund und OCT-Untersuchung stellten wir die Diagnose einer akuten makulären Neuroretinopathie (AMNR) des rechten Auges. Aufgrund des engen zeitlichen Zusammenhangs vermuteten wir, dass die Impfung gegen COVID-19 mit Comirnaty® von BioNTech/Pfizer die AMNR ausgelöst hat.

## Therapie und Verlauf

Da aktuell keine therapeutischen Optionen bekannt sind, vereinbarten wir zunächst eine Kontrolluntersuchung der Patientin in 4 Wochen.

Das Lesen der Anfangsbuchstaben eines Wortes war auch bei den Kontrolluntersuchungen unverändert erschwert. Das parazentrale Skotom am rechten Auge bestand weiterhin. Sowohl Visustestung als auch Amsler-Test und klinischer Untersuchungsbefund zeigten keine Veränderung im Vergleich zur Erstvorstellung der Patientin.

**Figure 4:**
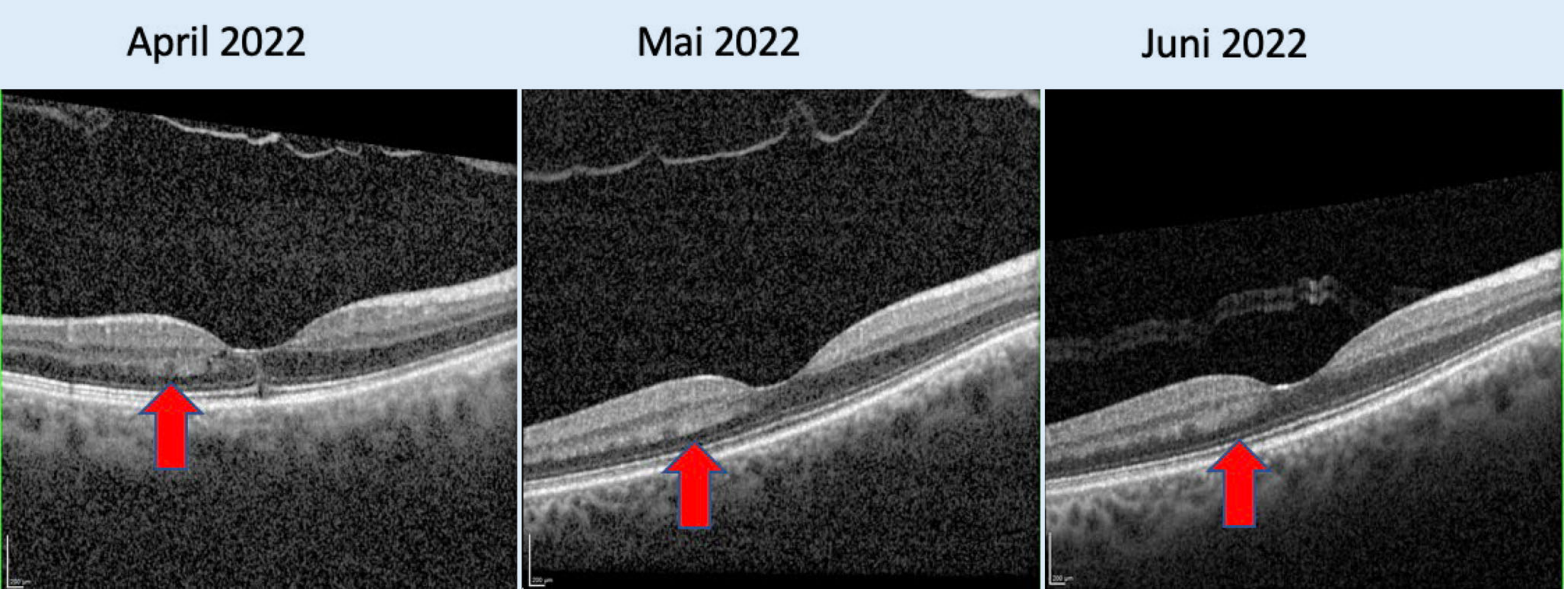

**Figure 5:**
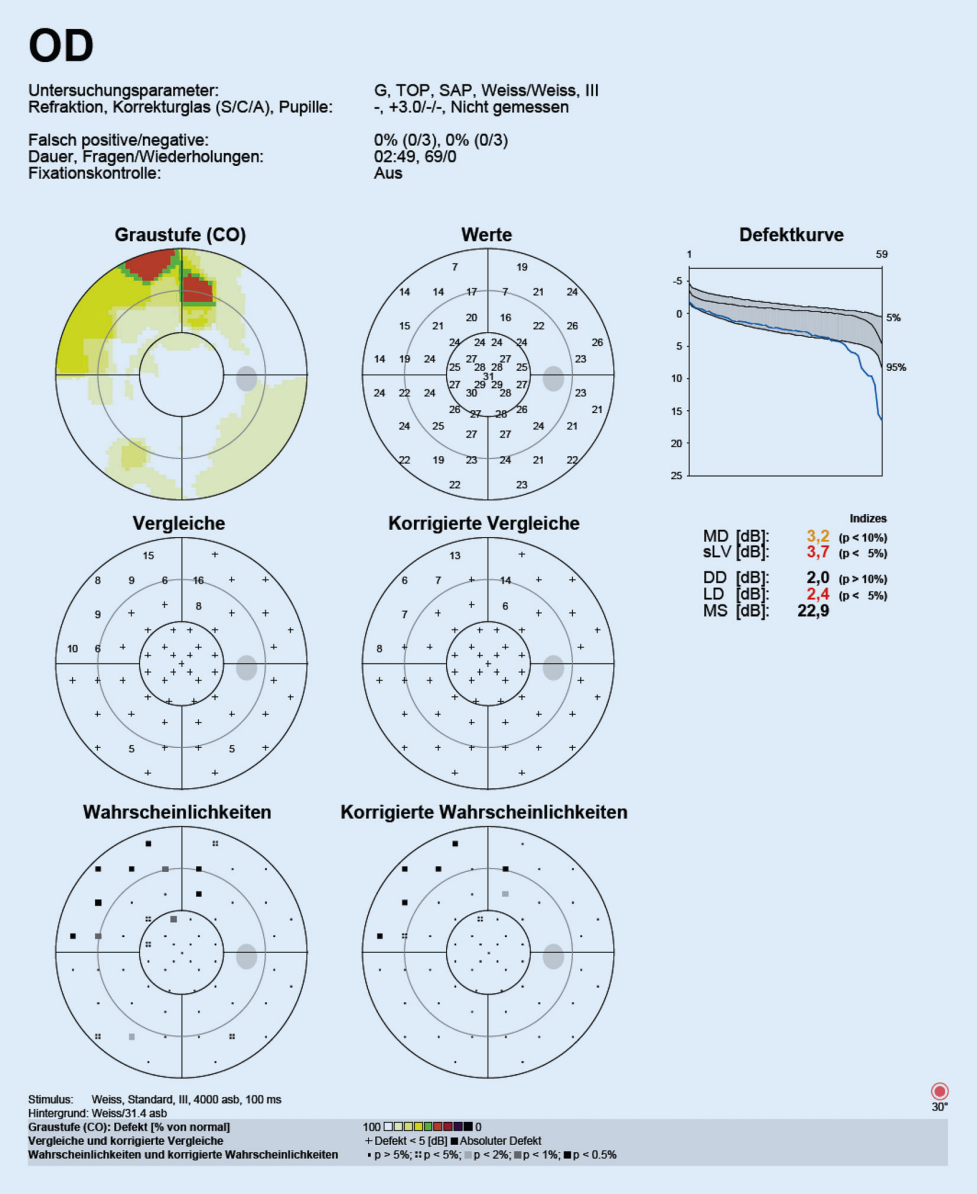

Hingegen stellten sich in der OCT-Untersuchung die für die AMNR typischen hyperreflektiven Plaques der äußeren plexiformen und äußeren Körnerschicht temporal der Fovea des rechten Auges deutlicher dar (Abb. 4). Fluoreszenzangiographie und Perimetrie blieben weiterhin unauffällig (Abb. 5).

## Diskussion

Die AMNR wurde erstmals im Jahr 1975 von Bos und Deutman als Erkrankung der oberflächigen Schichten der Retina beschrieben [3]. Sie äußert sich in Form von parazentralen Skotomen. Fundoskopisch zeigen sich dunkle, braun-rote Läsionen neben der Fovea. Betroffen waren v. a. junge Frauen, die eine orale Kontrazeption einnahmen.

In den letzten Jahren wurden weitere Auslöser der Erkrankung identifiziert (Tab. 1; [1]). Bis heute ist die Ätiologie der Erkrankung unklar. Vor allem mikrovaskuläre Ursachen werden diskutiert, wobei vermutet wird, dass eine nichtinflammatorische Ischämie des tiefen kapillären Plexus der Retina das typische Krankheitsbild verursacht [1, 7].

**Table 1:** 

Beispiele möglicher Auslöser einer AMNR	Literaturbeispiele
Grippaler Infekt, febriler Infekt, Infekt der oberen Atemwege, Influenza	[1]
Orale Kontrazeption	[1]
Epinephrin-Exposition	[1]
Trauma	[1]
Systemischer Schock	[1]
Dengue-Fieber	[10]
CMV-Infektion	[14]
COVID-19-Infektion	[17]
Impfung mit Vaxzevria® von AstraZeneca	[2]
Impfung mit Comirnaty von BioNTech/Pfizer	[9]

Die Veränderungen der äußeren Körnerschicht, der äußeren plexiformen Schicht und in fortgeschrittenen Fällen auch der IS/OS-Junctions und der ellipsoiden Zone können persistieren, sodass die subjektiv störenden parazentralen Skotome weiter wahrgenommen wurden [16]. Neuhann et al. beschreiben bei einigen Fällen einen spontanen Rückgang der Beschwerden nach einigen Monaten [12].

Hiervon abzugrenzen ist die parazentrale akute mittlere Makulopathie (PAMM). Sie zeichnet sich durch Hyperreflektivität der inneren Körnerschicht aus und wurde zunächst als Unterform der AMNR definiert [5, 9]. Da sie in Kombination mit zilioretinalen arteriellen Gefäßverschlüssen beschrieben wurde, wird eine Ischämie des mittleren und tiefen kapillären Plexus der Retina als ihre Ursache vermutet [5, 13].

Da bei unserer Patientin die hyperreflektiven Bande in der äußeren plexiformen und äußeren Körnerschicht lokalisiert sind, definieren wir ihr Krankheitsbild als AMNR nach COVID-19-Impfung, nicht als PAMM.

Im Zeitalter der COVID-19-Pandemie hat die Thematik der AMNR eine neue Brisanz erlangt. Zum einen werden Krankheitsfälle in direktem Zusammenhang mit einer COVID-19-Infektion beschrieben. Zum anderen gibt es einige Berichte von AMNR nach COVID-19-Impfung, insbesondere nach Impfung mit Vaxzevria® von AstraZeneca. Beinahe ausnahmslos waren junge Frauen betroffen [2, 6, 11].

Das Auftreten einer AMNR nach Impfung mit Comirnaty® von BioNTech/Pfizer scheint deutlich seltener zu sein. Lediglich vereinzelte Berichte aus Nashville, Japan und Sydney liegen diesbezüglich vor [4, 9, 18].

Als potenzielle Ursache der retinalen Veränderungen ist differenzialdiagnostisch die Immuntherapie der Patientin mit Trastuzumab und Pertuzumab in Betracht zu ziehen. Bisher sind allerdings keine Fälle einer AMNR durch Trastuzumab oder Pertuzumab bekannt geworden. Dennoch kann der immunmodulierende Effekt in Kombination mit der COVID-19-Impfung hinsichtlich der Entstehung einer AMNR nicht außer Acht gelassen werden.

Derzeit ist keine kausale Therapie der AMNR bekannt. Es gibt vereinzelte Berichte einer Verbesserung der tiefen kapillären und chorioidalen Blutversorgung nach oraler Prednisolon-Gabe [8]. Standardisierte Therapieoptionen können hieraus jedoch nicht abgleitet werden.

Im Hinblick auf zukünftige Behandlungsmöglichkeiten der AMNR ist ihre Prädilektionsmanifestation temporal der Fovea – vergleichbar mit makulären Teleangiektasien Typ II – hervorzuheben. Da die Gefäßarkade der Netzhaut im Bereich der temporalen Fovea ausgedünnt ist, wurde vermutet, dass die Blutversorgung temporal der Fovea im Vergleich zur restlichen Netzhaut schlechter ist und sich mikrovaskuläre Ischämien hier bevorzugt manifestieren („Prinzip der letzten Wiese“) [15].

## Fazit für die Praxis

- Die Anamneseerhebung ist die Grundlage der Diagnosestellung der AMNR.
- Liegen subjektive parazentrale Skotome vor, die insbesondere das Lesen einschränken, muss die AMNR differenzialdiagnostisch in Erwägung gezogen werden. Es sollte weitere apparative Diagnostik, v. a. mittels Makula-OCT erfolgen.
- Aufgrund des engen zeitlichen Zusammenhangs der COVID-19-Impfung und der Erkrankung unserer Patientin sowie der bisher publizierten Fallberichte kann die COVID-19-Impfung als Auslöser der AMNR angenommen werden.
